# Pediatric meningococcocal meningitis in the acute phase: how much does it cost?

**DOI:** 10.1186/s13052-019-0616-z

**Published:** 2019-02-13

**Authors:** Elena Bozzola, Stefano Guolo, Enea Bonci, Chiara Rossetti, Mauro Bozzola, Massimiliano Raponi, Alberto Villani

**Affiliations:** 1 Pediatric and Infectious Disease Unit, Bambino Gesù Children Hospital, Rome, Italy; 2 Sanitary Direction, Bambino Gesù Children Hospital, Rome, Italy; 3 Sperimental Medicine Department, La Sapienza University, Rome, Italy; 4 Internal Medicine and Therapeutics Department, Pediatrics and Adolescentology Unit, University of Pavia, Pavia, Italy

**Keywords:** Meningitis, Hospital, Cost, Children

## Abstract

**Background:**

Meningococcal meningitis (MM) is known to be responsible of high cost for the Public Health Administration. Aim of the work is to calculate the costs for the hospitalization of pediatric patients affected by MM.

**Methods:**

We calculate the costs for the hospitalization of pediatric patients affected by MM in the acute phase (HAP) over a nine year period. We performed a MEDLINE search to verify the cost of MM HAP reported in other studies.

**Results:**

At Bambino Gesù Children Hospital, the median cost of HAP was of 12,604 euro (range from 9203 to 35,050 euro). Comparing our data with the previous studies, we find out similar results of approximately 16,750 euro (range 12,000–20,000 euro).

**Discussion:**

Despite the relative rarety of the disease, MM is associated to direct high cost of HAP.

**Conclusions:**

Hospital costs are an important end-point in health economic evaluation of the disease and may be useful to policy makers and health economists to understand the potential benefit of improving meningococcal vaccination programmes.

## Background

Neisseria meningitidis (Nm) is the causative agent of meningococcal meningitis (MM).

Among the 13 subgroups of Nm, six of them (A,B,C,W135,X,Y) may cause clinical disease in humans and may be responsible of mortality and morbidity among survivors. [1–3]

In Italy, the incidence of MM is of 0.3 cases per year per 100,000 habitants, according to the Italian National Institute of Public Health. In the pediatric age, the highest incidence is among children aged less than 4 years. In particular, in infants younger than 1 year, 4 cases per year per 100,000 had been notified. [4]

MM is also responsible of high cost for the acute care and for the follow-up of affected patients. [5–7] To date, vaccination constitutes the best preventive strategies against MM and it can protect against subgroups A, B, C, Y and W135. In Italy, antimeningoccocal vaccines are currently included in the routine childhood vaccination strategies, but they are not compulsory. [8] The first meningococcal conjugate vaccine was against serogroup C and was introduced in the Italian national immunization plan in 2005. A significant reduction in the incidence of invasive meningococcal disease in the post vaccination period compared to the pre-vaccination one has been observed in Italy. [9, 10] The actual vaccination coverage against Nm C is of 80,67% at 24 months of life. The coverage against Nm B is very low as just 14,72% of 2 years old children received the immunization. Finally, 18,28% of 2 years old children received the vaccine against Nm ACW135Y.

Aim of the study is to estimate the costs of MM hospitalization in the acute phase (HAP) in an Italian pediatric population.

## Material and methods

The study was performed at Bambino Gesù Children Hospital, which is a a tertiary pediatric hospital in Rome, Lazio, Italy. The records of patients aged < 18 years admitted at hospital for MM were analyzed from January 2006 till January 2015. The MM cases were identified using the international classification of diseases, ninth revision, clinical modification (ICD-9-CM) which assigns numeric codes to diagnosis. ICD-9 code which identifies MM is 036.0.

The medical records of patients with principal discharge ICD-9-CM code 036.0 were analyzed in order to verify the laboratory-confirmed diagnosis. If the cerebrospinal fluid antigen and/or the culture was positive for Nm, the patient entered the study.

Patients who did not fulfill the inclusion criteria were excluded from the study. As for the others, direct medical costs were extracted from the Lazio Regional Health Service Tariffs and included: cost of hospital accommodation and management at the Infectious Diseases Unit and/or at the Intensive care Unit. An important part of hospitalization is caused by the cost of each single day of hospitalization. The price per day of hospital stay is different between the Departments of Infectious Diseases and of Intensive Care. This is due to the strong difference in care complexity of the two departments. We considered a daily cost of € 626 for the intensive care unit, defined as a very high complexity unit and € 476 for the infectious diseases, defined as a low complexity unit.

To this cost, the price of procedures (imaging, laboratory exams, medical and paramedical evaluations), medical and surgical treatment were added. The appropriate procedure codes were applied, in order to evaluate the single cost of any exam and therapy.

We performed a MEDLINE search to calculate the cost of MM HAP. For the purpose of this study, we used the keywords “Meningitis, Meningococcal”[Mesh] AND “Costs and Cost Analysis”[Mesh] OR “Hospital Costs”[Mesh] OR “Employer Health Costs”[Mesh] OR “Health Care Costs”[Mesh] OR “Drug Costs”[Mesh] OR “Cost of Illness”[Mesh] OR “Direct Service Costs”[Mesh] OR “Health Expenditures”[Mesh] OR “Cost-Benefit Analysis”[Mesh] OR “Economics”[Mesh]. The search concerned English publications referring to patients younger than 18 years of age, from January 2006 to December 2016.

We included in the search cases of pediatric laboratory-confirmed MM with precise cost of HAP. We excluded reports: [1] if they concerned meningitis with undistinguished or unconfirmed etiology; [2] if they concerned adults; [3] if they presented undistinguished data on both adults and children; [4] if they report the undistinguished cost of HAP and of follow-up.

From all reports, we extracted both the number of MM and the mean cost of HAP.

## Results

Among all the children hospitalized in the study period, 32 fulfilled the inclusion criteria. The mean age of patients was of 5.3 years (range from 40 days to 16.5 years, median 4 years). The proportion of male and female was similar (53% males and 47% female). All the patients had neither an underlying medical condition nor received previous anti-meningococcal vaccination.

Among enrolled patients, subgroup C has been isolated in 7 cases, subgroup B in 9, subgroup Y in 3 and subgroup W135 in 1 patient. In 12 cases no subgroup has been detached.

The median cost of HAP was of 12,604 euro (range from 9203 to 35,050 euro; mean cost of 14,874 euro).

Medical condition both at MM onset and during hospitalization may have complicated the disease, requiring a prolonged length stay, further exams and therapies.

Table 1 summarizes the costs for each patients.

**Table 1:** HAP cost for each patients (in euro)

Patient	SEX	AGE	HAP costs	Intensive care costs	Laboratory costs	Instrumental costs	Specialist visits costs	Therapy costs
1	F	0,09	9203	0	843	223	21	26
2	F	9,9	9475	0	671	167	41	122
3	M	3,98	9533	1236	865	207	62	69
4	M	3,39	9545	0	683	243	41	42
5	M	9,82	9638	1050	761	144	41	136
6	F	1,92	9740	0	874	342	41	196
7	F	0,56	10,094	0	871	309	62	53
8	F	8,25	10,224	0	689	266	41	84
9	F	7,28	10,320	0	689	278	83	101
10	M	1,27	10,372	0	817	509	41	66
11	M	3,99	10,930	741	947	179	124	104
12	M	6,6	11,526	0	816	412	165	83
13	F	0,53	11,565	0	944	200	41	35
14	F	1,09	11,696	2317	979	98	83	43
15	M	15,46	11,708	0	748	98	41	148
16	F	0,8	11,958	0	832	243	41	32
17	F	16,51	13,250	3274	816	278	83	319
18	M	15,3	13,783	2224	962	290	83	181
19	F	1,26	14,528	2749	1016	332	83	433
20	F	1,99	14,809	0	835	230	83	86
21	M	11,22	15,069	3700	936	274	62	141
22	F	4,01	15,267	0	748	266	83	108
23	M	1,23	15,595	0	689	573	41	41
24	M	2,09	15,757	0	959	278	83	132
25	M	11,67	16,486	2440	827	1130	248	259
26	M	5,23	16,754	0	746	972	145	83
27	M	0,6	21,022	0	869	713	269	76
28	M	2,36	21,993	2224	634	376	62	7659
29	F	0,5	26,309	5838	1212	407	186	3260
30	F	9,86	26,352	2687	1085	572	207	9034
31	M	6,06	26,423	5190	1014	278	124	9329
32	M	7,19	35,050	4448	1050	584	124	13,542

The mean HAP of the six patients aged < 1 year was of 17,306 euro, higher than those of the 12 children aged 1 to 5 years (13,313 euro), as well as of the eight cases aged 5 to 10 years (15,025 euro), and of the six patients older than 10 years (14,059 euro).

Patients had been hospitalized for a mean time of 17 days (range from 11 to 25 days). Fourteen children required Intensive care Unit assistance for a mean period of 3 days (range from 24 to 189 h), which contributes to the increment of the hospitalization costs.

Patients requiring Intensive Unit Care had higher HAP (17,931 euro) than those hospitalized at Infectious Diseases Unit (12,496 euro).

The HAP during the period study did not change.

Out of 16 reports resulting from our MEDLINE analysis, just 2 manuscripts matched the criteria for inclusion in the study. No Italian manuscript matched the inclusion criteria. The manuscript were carefully examined to estimate HAP. [5, 6]

The other 14 manuscripts were excluded because they: [1] concern costs on vaccine and vaccination programmes [7]; [2] report costs only on diagnostic tools [1]; [3] reported indistinguishable data on the adult and pediatric populations [2]; [6] reported only clinical data [1]; [7] reported not real cost of HAP but scenario [2, 8] reported data from other sources [1].

Comparing our data with the selected studies, we find out similar results of approximately 16,750 euro (range 12,000–20,000 euro). [6, 7]

## Discussion

In our study, we reported the cost of MM HAP. Hospital costs are an important end-point in health economic evaluation of the disease.

We analyzed cost associated with inpatient servicies in the acute phase which are one of the major component of health care utilization in the management of MM. [11, 12]

The cost of the acute phase of the disease with a high morbidity among survivors may constitute the tip of the iceberg of the disease. [5]

We demonstrated that MM is associated with direct health cost as for a median of 12,604 euro each patient. Despite the relative rarety of MM, the high cost of HAP translate to a consistent economic burden. In fact, we have also to consider indirect costs, such as missing working days for parents who assist children (productivity loss) and of prophylaxis to close contacts of the index case, to prevent outbreaks. Moreover, also medical and paramedical equipment who should be instructed to manage MM represent an indirect cost.

Numerous studies estimating the costs of meningococcal disease have previously been performed. Nevertheless most studies used ICD diagnosis codes not verified against laboratory results or hospital records. [11, 12] A possible consequence is an under or over estimation of hospital costs. Moreover, other studies did not differentiate costs among the acute phase and lifelong costs of treatment and management of invalidating sequelae. [5] Few studies are available only on MM HAP. [6, 7]

Potential limits of our study is the small sample size and that all data were collected from a single pediatric institution. Finally, a limitation of the comparing analysis is that the previous works did not report the single cost items, so that the result is approximate and may be influenced by a different approach to acute patient.

## Conclusions

In conclusion, we conducted this analysis as we think that financial studies are important to evaluate the cost saving in order to public funding decisions like immunization strategies and vaccination programmes. Economic evaluation, including length of hospitalization, exams and treatments, may be useful to policy makers and health economists to understand the potential benefit of improving meningococcal vaccination programmes.

## Abbreviations

HAP: hospitalization in the acute phase
MM: meningococcal meningitis
Nm: Neisseria meningitidis
